# A Randomized Trial of Encorafenib and Cetuximab Versus Irinotecan/Cetuximab or FOLFIRI/Cetuximab in Chinese Patients With BRAF^V600E^ Mutant Metastatic Colorectal Cancer: The NAUTICAL Study

**DOI:** 10.1002/cam4.71697

**Published:** 2026-03-19

**Authors:** Wang Xicheng, Deng Yanhong, Zhang Yanqiao, Liu Tianshu, Yuan Xianglin, Yang Jianwei, Zhang Tao, Zang Aimin, Liu Yu, Huang Li, Ye Feng, Zong Hong, Ba Yi, Klauck Isabelle, Vedovato Jean‐Claude, Groc Mélanie, Guo Angela, Li Jian, Shen Lin

**Affiliations:** ^1^ Peking University Cancer Hospital and Institute Beijing China; ^2^ Sun Yat‐Sen University Guangzhou Guangzhou China; ^3^ Harbin Medical University Cancer Hospital Harbin Harbin China; ^4^ Zhongshan Hospital Fudan University Shanghai China; ^5^ Department of Oncology, Tongji Hospital, Tongji Medical College Huazhong University of Science and Technology Wuhan China; ^6^ Fujian Cancer Hospital Fuzhou China; ^7^ Affiliated Hospital of Hebei University Baoding Baoding China; ^8^ The Second People's Hospital of Neijiang Neijiang China; ^9^ First Affiliated Hospital of Gannan Medical University Ganzhou China; ^10^ The First Affiliated Hospital of Xiamen University Xiamen China; ^11^ The First Affiliated Hospital of Zhengzhou University Zhengzhou China; ^12^ Tianjin Medical University Cancer Institute and Hospital Tianjin China; ^13^ Pierre Fabre Médicament Boulogne‐Billancourt France; ^14^ Pierre Fabre Laboratories Beijing China

**Keywords:** BRAF^V600E^, cetuximab, China, colorectal cancer, encorafenib, progression‐free survival

## Abstract

**Background:**

Colorectal cancer (CRC) is a major health burden globally and in China, where 3%–5% of cases involve the BRAF^V600E^ mutation, which is associated with aggressive disease and therefore a poor prognosis. Although the combination of encorafenib and cetuximab has demonstrated improved survival in BRAF^V600E^ mutant metastatic CRC (mCRC), such treatments remain unavailable as chemotherapy‐free options in China.

**Methods:**

The NAUTICAL CRC, a Phase II study in Chinese patients with BRAF^V600E^ mutant metastatic CRC, includes a Safety Lead‐In (SLI) phase for tolerability assessment and a randomized phase comparing encorafenib/cetuximab vs. irinotecan‐based regimens, potentially bridging the treatment gap and evaluating safety, efficacy, and Quality of Life (QoL) outcomes in this Chinese population.

**Results:**

No dose‐limiting toxicity was identified in the SLI phase (*N* = 10). In the Randomized phase (*N* = 97), the Doublet arm demonstrated superior progression‐free survival (PFS) of 4.2 months vs. 2.5 months in the Control arm (hazard ratio [HR]: 0.37, *p* = 0.0004) and longer overall survival (OS) of 11.6 months vs. 8.2 months (HR: 0.55). Treatment‐related adverse events were common but more severe in the Control arm. QoL measures consistently favored the Doublet arm, showing improved health status and reduced deterioration risk.

**Conclusions:**

This NAUTICAL CRC Phase II study showed that the combination of encorafenib and cetuximab offers significant clinical benefits, improving PFS and OS, while providing manageable safety and important QoL advantages, making it a valuable treatment option for Chinese patients with previously treated BRAF^V600E^ mutant mCRC.

**Trial Registration:**

Clinical study registration number: NCT05004350

## 1 Introduction

Colorectal cancer (CRC) is one of the most prevalent cancers worldwide, with its incidence steadily rising over recent decades [1]. In 2022, CRC accounted for 517,000 new cases and 240,000 deaths in China, making it the second most common cancer and the fourth leading cause of cancer‐related mortality in the Chinese population [2]. A subset of CRC cases, approximately 3%–5% in China, is driven by mutations in the B‐RAF proto‐oncogene, serine/threonine kinase (BRAF), which leads to aberrant activation of the mitogen‐activated protein kinase (MAPK) pathway [3, 4]. This activation promotes uncontrolled cell proliferation and survival, contributing to poor clinical outcomes, particularly in patients with metastatic colorectal cancer (mCRC) harboring the BRAF^V600E^ mutation [5, 6]. BRAF^V600E^ mutant mCRCs exhibit an unfavorable and heterogeneous prognosis, as patients tend to have poorer responses to chemotherapy regimens [7, 8]. First‐line intensive chemotherapy does not significantly improve progression‐free survival (PFS) or overall survival (OS) in Chinese patients with BRAF^V600E^ mutant mCRC, highlighting the urgent need for novel therapeutic approaches to enhance outcomes in this population [4, 9].

Encorafenib, a highly selective adenosine triphosphate (ATP)‐competitive RAF kinase inhibitor, was developed to target tumors with BRAF^V600^ mutations [10, 11]. By inhibiting the MAPK pathway, encorafenib has demonstrated potential across various BRAF^V600^ mutant tumor types, including melanoma, lung, and colorectal cancers [10]. In mCRC, BRAF inhibitors alone are not effective due to the feedback activation of epidermal growth factor receptor (EGFR) in BRAF‐mutant CRC, which leads to continued cell proliferation [12]. This feedback can be overcome by simultaneously targeting multiple nodes in the pathway, such as EGFR, thereby enhancing the therapeutic efficacy of BRAF inhibition [13]. In mCRC specifically, the combination of encorafenib with the EGFR inhibitor cetuximab has been a major focus of clinical investigation [14].

The pivotal Phase III BEACON CRC study (NCT02928224) evaluated this combination of encorafenib and cetuximab with or without the mitogen‐activated protein kinase inhibitor binimetinib in patients with BRAF^V600E^ mCRC whose disease had progressed after one or two prior therapies [15, 16]. Results from the BEACON study showed significant improvements in OS and objective response rate (ORR) with both the doublet (encorafenib and cetuximab) and triplet (encorafenib, cetuximab, and binimetinib) regimens compared to standard chemotherapy [17, 18]. The more favorable safety profile and overall better benefit–risk ratio of the doublet led to its approval in the United States and Europe in 2020 as a treatment option for patients with BRAF^V600E^ mCRC who had failed after prior systemic therapy [17, 18].

However, in China, the treatment landscape for BRAF^V600E^ mCRC remains limited, and no chemotherapy‐free treatment regimen is yet available for this patient population [19]. To address this gap, the NAUTICAL CRC study (NCT05004350) was designed as a Phase II, multicenter, randomized, open‐label study to evaluate the efficacy and safety of the encorafenib and cetuximab doublet in Chinese patients with BRAF^V600E^ mCRC who had progressed after one or two prior treatments [20]. This study serves as a bridging study to confirm the consistency of the treatment effect observed in the BEACON CRC study within the Chinese population.

Here, we present the efficacy and safety results of the NAUTICAL CRC Phase II study, which compares the encorafenib and cetuximab doublet to the investigator's choice of control treatment (irinotecan/cetuximab or FOLFIRI/cetuximab) in Chinese patients with BRAF^V600E^ mCRC. These findings provide critical insights into the applicability of this targeted therapy approach in a population where therapeutic options remain limited.

## 2 Research Design and Methods

### 2.1 Study Design and Patients

NAUTICAL CRC is a Phase II, multicenter, randomized, open‐label, two‐arm comparative study evaluating doublet (encorafenib/cetuximab) vs. control (irinotecan/cetuximab or FOLFIRI/cetuximab) in Chinese patients with BRAF^V600E^ mutant mCRC whose disease progressed after one or two prior treatment lines in the metastatic setting [20]. The study was approved by the institutional review board or independent ethics committee at each center and was conducted in accordance with the requirements of the regulatory authorities and with the provisions of the Declaration of Helsinki and the Good Clinical Practice guidelines of the International Council on Harmonization. All patients provided written informed consent.

The study contains a safety lead‐in (SLI) phase to assess the preliminary safety and tolerability of the doublet in Chinese patients prior to enrolling patients in the Randomized phase (Figure S1). The SLI phase was designed to recruit a limited number of patients and was conducted at one study site. A total of nine patients were planned to be assigned to treatment (encorafenib 300 mg, per os [po, orally] once daily (QD) AND cetuximab 400 mg/m^2^ intravenous [IV] 120 min then 250 mg/m^2^ IV 60 min, once a week [QW]) on a rolling basis in a single cohort and received 28‐day cycles of treatment. An Independent Data Monitoring Committee reviewed both the dose‐limiting toxicities (DLTs) and cumulative toxicity to evaluate safety and tolerability. Within the Randomized phase 94 patients were overall planned to be randomized in a 2:1 ratio to the doublet arm or control arm. Randomization was stratified by baseline Eastern Cooperative Oncology Group (ECOG) performance status (0 vs. 1) and prior use of irinotecan (yes vs. no).

Patients in the doublet arm received encorafenib (300 mg, po QD) AND cetuximab (400 mg/m^2^ IV 120 min then 250 mg/m^2^ IV 60 min, QW). Patients in the control arm received irinotecan (180 mg/m^2^ IV 90 min, once every 2 weeks [Q2W]) AND cetuximab (400 mg/m^2^ IV 120 min then 250 mg/m^2^ 60 min, QW) OR FOLFIRI (irinotecan [180 mg/m^2^ IV 90 min Q2W]; folinic acid [400 mg/m^2^ IV, 120 min Q2W]; 5‐fluorouracil [400 mg/m^2^ IV 15 min then 1200 mg/m^2^/day × 2 days, Q2W] AND cetuximab [400 mg/m^2^ IV 120 min then 250 mg/m^2^ 60 min, QW]). The decision to treat with 5‐fluorouracil/folinic acid + irinotecan (FOLFIRI) or irinotecan in the control arm was made solely at the discretion of the investigator and reflected contemporaneous guidance [21, 22]. Patients were treated until disease progression, unacceptable toxicity, or consent withdrawal.

Participants were assessed every 6 weeks (±7 days) from the date of randomization for the first 24 weeks, then every 12 weeks (±7 days) thereafter until disease progression or death, withdrawal of consent, initiation of subsequent anticancer therapy, loss to follow‐up, or study end, whichever occurred first.

The study started in China with enrollment of the first SLI patient in September 2021, and recruitment concluded with the enrollment of the last patient in April 2023. The primary completion date was reached in December 2023. A total of 30 sites were enrolling patients across the country. The NAUTICAL Study conduct partially coincided with the coronavirus disease 2019 (COVID‐19) pandemic.

### 2.2 Study Participants

Adult participants with BRAF^V600E^ mutant mCRC who progressed after one or two prior treatment lines in the metastatic setting were eligible for the NAUTICAL study if they had measurable disease according to RECIST v1.1 criteria, an ECOG performance status of 0 or 1, and adequate hematologic, hepatic, and renal function. For further details, please see the Methods in Appendix S1.

### 2.3 Study Endpoints

The primary endpoint of the SLI phase was to assess the safety and tolerability of the doublet therapy, which was measured by the incidence of DLTs occurring during the DLT‐evaluation period, defined as the first 28 days after the initial dose of the study intervention. Secondary endpoints included the overall safety profile, preliminary pharmacokinetics (PK) (data not presented), and antitumor activity.

The primary endpoint of the Randomized phase was Progression‐Free Survival (PFS), assessed by blinded independent central review (BICR). Secondary endpoints included PFS assessed by the investigator, Objective Response Rate (ORR), Duration of Response (DOR), Disease Control Rate (DCR), and Time to Response (TTR), all of which were evaluated by both BICR and the investigator. Additional secondary endpoints included Overall Survival (OS), safety and tolerability, and quality of life (QoL) as measured by patient‐reported outcomes (PROs). These outcomes were assessed using the European Organization for Research and Treatment of Cancer Quality of Life Questionnaire‐Core 30 (EORTC QLQ‐C30), the EuroQoL 5‐Dimension 5‐Level (EQ‐5D‐5L), the Functional Assessment of Cancer Therapy‐Colon Cancer (FACT‐C), and the Patient Global Impression of Change (PGIC). Definitions of endpoints are available in the Methods in Appendix S1.

As a component of the secondary endpoints, PK data were obtained through serial blood sampling to measure the plasma concentrations of encorafenib and cetuximab (data not presented). Mismatch repair status is a tertiary objective and will be part of the final analysis.

### 2.4 Statistical Analysis

The study's sample size was calculated to detect a PFS benefit, aiming for a median difference of 2 months between treatment arms, from 2 to 4 months, corresponding to a hazard ratio (HR) of 0.5 with a one‐sided log‐rank test (alpha = 0.025 and beta = 0.2).

Baseline characteristics were summarized using descriptive statistics and proportions by treatment arm. Time‐to‐event endpoints, DCR, ORR, and PROs were analyzed using the Full Analysis Set (FAS), which included all randomized patients. Safety was evaluated by assessments of adverse events (AEs) and laboratory abnormalities in patients who received at least one dose of study drug (Safety Set [SAF]).

Formal statistical testing was performed on the primary analysis of the primary endpoint in the Randomized phase (all other statistical results are to be considered as descriptive).

For the primary analysis of PFS, nonparametric statistics using the Kaplan–Meier method were employed to calculate the median, with 95% confidence intervals (CIs) provided using the Brookmeyer–Crowley method. Comparisons between the two treatment arms were performed using a one‐sided stratified log‐rank test in the Randomized phase, with an overall significance level set at 0.025. A Cox regression model stratified by randomization strata was used to estimate the HR and the corresponding 95% CIs, based on the Wald test. A similar approach was taken for the other time‐to‐event efficacy endpoints (OS, DOR, and TTR).

For the primary efficacy criterion, a multivariate Cox proportional hazards model was also used to assess the effect of confounding variables on PFS, using ECOG performance status (0 vs. 1) and prior irinotecan use (yes vs. no) as stratification factors, with a nominal *p*‐value of < 0.05.

PFS subgroup analyses were conducted for each baseline stratification factor and other relevant baseline variables. No inferential procedure was implemented on subgroup analyses; subgroups were analyzed in a descriptive manner. They are presented in forest plots comparing the Doublet arm and Control arm, with unstratified HRs and 95% CIs. Subgroups were analyzed when at least 10 events were reported within the given subgroup. The ORR was provided with a corresponding Clopper–Pearson (exact) binomial 95% CI.

For further details, please see the Methods in Appendix S1.

All statistical analyses were performed using the Statistical Analysis System (SAS), version 9.4.

## 3 Results

### 3.1 SLI Phase

Patients in the SLI phase (*N* = 10) were enrolled between September 3, 2021 and November 5, 2021. As of the data cut‐off (December 19, 2023), one SLI patient continued to receive treatment (Figure S2).

Enrolled patients had a mean (SD) age of 57.90 (13.15) years, equal distribution of left and right colon cancers, a baseline performance status of 70.0% with ECOG 0, higher rates of complete tumor resection (90.0%), and greater metastatic involvement in the lung (60.0%) and peritoneum (60.0%). Additionally, most patients had elevated carcinoembryonic antigen (CEA) levels > 5 μg/L (80.0%), elevated cancer antigen 19‐9 (CA 19‐9) levels > 35 IU/mL (70.0%), and 60.0% had C‐reactive protein (CRP) levels > 0.01 g/L (Table 1).

**TABLE 1:** Baseline characteristics (FAS).

	SLI phase	Randomized phase		
	Doublet (*N* = 10)	Doublet (*N* = 65)	Control (*N* = 32)	Total (*N* = 97)
Sex, *n* (%)				
Male	7 (70.0)	34 (52.3)	11 (34.4)	45 (46.4)
Female	3 (30.0)	31 (47.7)	21 (65.6)	52 (53.6)
Age, mean (SD)	57.90 (13.15)	53.52 (14.00)	54.56 (14.78)	53.87 (14.19)
Age, *n* (%)				
< 65 years	6 (60.0)	49 (75.4)	19 (59.4)	68 (70.1)
65–74 years	4 (40.0)	10 (15.4)	13 (40.6)	23 (23.7)
≥ 75 years	0	6 (9.2)	0	6 (6.2)
Primary site of cancer, *n* (%)				
Colon, left	5 (50.0)	39 (60.0)	16 (50.0)	55 (56.7)
Colon, right	5 (50.0)	26 (40.0)	16 (50.0)	42 (43.3)
ECOG PS at baseline, *n* (%)				
0	7 (70.0)	22 (33.8)	9 (28.1)	31 (32.0)
1	3 (30.0)	43 (66.2)	23 (71.9)	66 (68.0)
Time since initial diagnosis, months, mean (SD)	14.29 (14.15)	14.15 (13.28)	15.63 (11.38)	14.64 (12.64)
Removal status of primary tumor, *n* (%)				
No resection	1 (10.0)	16 (24.6)	5 (15.6)	21 (21.6)
Partial resection	0	8 (12.3)	6 (18.8)	14 (14.4)
Complete resection	9 (90.0)	41 (63.1)	21 (65.6)	62 (63.9)
Number of prior metastatic line, *n* (%)				
1	4 (40.0)	50 (76.9)	25 (78.1)	75 (77.3)
2	6 (60.0)	15 (23.1)	7 (21.9)	22 (22.7)
Metastatic disease site (> 40%), *n* (%)				
Liver	4 (40.0)	39 (60.0)	16 (50.0)	55 (56.7)
Lung	6 (60.0)	26 (40.0)	16 (50.0)	42 (43.3)
Lymph node	3 (30.0)	30 (46.2)	14 (43.8)	44 (45.4)
Peritoneum/omentum	6 (60.0)	27 (41.5)	11 (34.4)	38 (39.2)
Number of organs involved, *n* (%)				
≤ 2	4 (40.0)	30 (46.2)	20 (62.5)	50 (51.5)
≥ 3	6 (60.0)	35 (53.8)	12 (37.5)	47 (48.5)
Carcinoembryonic antigen, *n* (%)				
≤ 5 μg/L	2 (20.0)	13 (20.0)	8 (25.0)	21 (21.6)
> 5 μg/L	8 (80.0)	52 (80.0)	24 (75.0)	76 (78.4)
Cancer antigen 19‐9, *n* (%)				
≤ 35 IU/mL	3 (30.0)	19 (29.2)	10 (31.3)	29 (29.9)
> 35 IU/mL	7 (70.0)	46 (70.8)	22 (68.8)	68 (70.1)
C‐reactive protein, *n* (%)				
≤ 0.01 g/L	4 (40.0)	46 (73.0)	23 (74.2)	69 (73.4)
> 0.01 g/L	6 (60.0)	17 (27.0)	8 (25.8)	25 (26.6)
Missing	0	2	1	3

Abbreviations: ECOG, Eastern Cooperative Oncology Group; FAS, full analysis set; *n*, number of patients with the characteristic; *N*, number of patients; PS, performance status; SD, standard deviation; SLI, safety lead‐in.

In the SLI phase, no DLT was identified in the Chinese cohort, and the Randomized phase of the study started at the confirmed dose of encorafenib 300 mg QD in combination with cetuximab, according to standard dose and regimen (Table 2). In detail, the median duration of exposure was 16.5 weeks (range 4–109.7 weeks). Within the median DOR of 12.5 months, no patients achieved a Complete Response (CR), while 30.0% achieved Partial Response (PR), Stable Disease, or Progressive Disease (PD) (each *n* = 3; 95% CI: 6.7–65.2). The ORR was 30.0% (95% CI: 6.7–65.2).

**TABLE 2:** Tumor response in patients with BRAF^V600E^–mutant metastatic colorectal cancer by treatment arm as assessed by blinded independent central review (FAS).

	SLI phase	Randomized phase	
	Doublet (*N* = 10)	Doublet (*N* = 65)	Control (*N* = 32)
Duration of exposure, weeks			
Mean (SD)	27.21 (30.45)	23.48 (15.64)	9.51 (6.37)
Min/max	4.0/109.7	1.0/68.0	2.0/22.6
Best overall response, % (95% CI)			
Complete response (CR)	0.0 (0.0–30.8)	3.1 (0.4–10.7)	3.1 (0.1–16.2)
Partial response	30.0 (6.7–65.2)	21.5 (12.3–33.5)	3.1 (0.1–16.2)
Stable disease	30.0 (6.7–65.2)	50.8 (38.1–63.4)	21.9 (9.3–40.0)
Progressive disease (PD)	30.0 (6.7–65.2)	7.7 (2.5–17.0)	21.9 (9.3–40.0)
Non‐CR/non‐PD	0.0 (0.0–30.8)	1.5 (0.0–8.3)	0.0 (0.0–10.9)
Not evaluable (NE)	10.0 (0.3–44.5)	15.4 (7.6–26.5)	50.0 (31.9–68.1)
Objective response rate, % (95% CI)	30 (6.7–65.2)	24.6 (14.8–36.9)	6.3 (0.8–20.8)
Disease control rate, % (95% CI)	/	75.4 (63.1–85.2)	28.1 (13.7–46.7)
Duration of response, median months (95% CI)	12.5 (NE; NE)	8.2 (2.8; NE)	NE (NE; NE)^b^
Time to response^a^, median months (95% CI)	/	1.4 (1.3–1.6)	1.5 (1.4; NE)

Abbreviations: BRAF^V600E^, B‐Rapidly accelerating fibrosarcoma (BRAF) with a mutation of the BRAF gene in which valine (V) is substituted by glutamic acid (E) at amino acid 600; CI, confidence interval; FAS, full analysis set; *N*, number of patients; NE, not evaluable; SD, standard deviation; SLI, safety lead‐in.

^a^ For responders only.

^b^ Only two participants had a response and the duration of response could not be calculated.

### 3.2 Randomized Phase

#### 3.2.1 Patient Disposition and Baseline Characteristics

Patients in the randomized phase (*N* = 97, FAS) were enrolled between February 9, 2022 and April 25, 2023. Thereby, 65 patients were enrolled in the doublet arm and 32 in the control arm (13 received the combination irinotecan + cetuximab and 14 received FOLFIRI + cetuximab). Five patients in the control arm withdrew consent before first administration (*N* = 92, SAF) (Figure S2). At the data cut‐off, 8 patients in the doublet arm and one in the SLI cohort continued to receive treatment. The most common reason for discontinuation from study treatment was PD (69.1%), with a higher proportion in the doublet arm than the control arm (76.9% vs. 53.1%). Of the 10 randomized patients who withdrew their consent during the treatment period, one (1.5%) was from the doublet arm and nine (28.1%) were from the control arm. Out of these nine patients, five never received study treatment. At the data cut‐off, 27 randomized patients (27.8%) continued to be in survival follow‐up after treatment discontinuation, with similar proportions in the doublet arm (29.2%) and in the control arm (25.0%). Death was the main reason for discontinuation of survival follow‐up (49 deaths out of the 54 randomized patients having entered the follow‐up period and then discontinued).

In the Randomized phase, slightly more females (53.6%) were enrolled, and most patients were under 65 years (70.1%); the primary cancer site was the left colon in 56.7% of patients, 48.5% had metastases in three organs or more, 56.7% had liver metastases, and 77.3% received one prior metastatic treatment. Additionally, most patients had elevated CEA levels > 5 μg/L (78.4%), elevated CA 19‐9 levels > 35 IU/mL (70.1%), and 73.4% had CRP levels ≤ 0.01 g/L (Table 1).

#### 3.2.2 Efficacy

##### 3.2.2.1 Progression‐Free Survival

**FIGURE 1:**
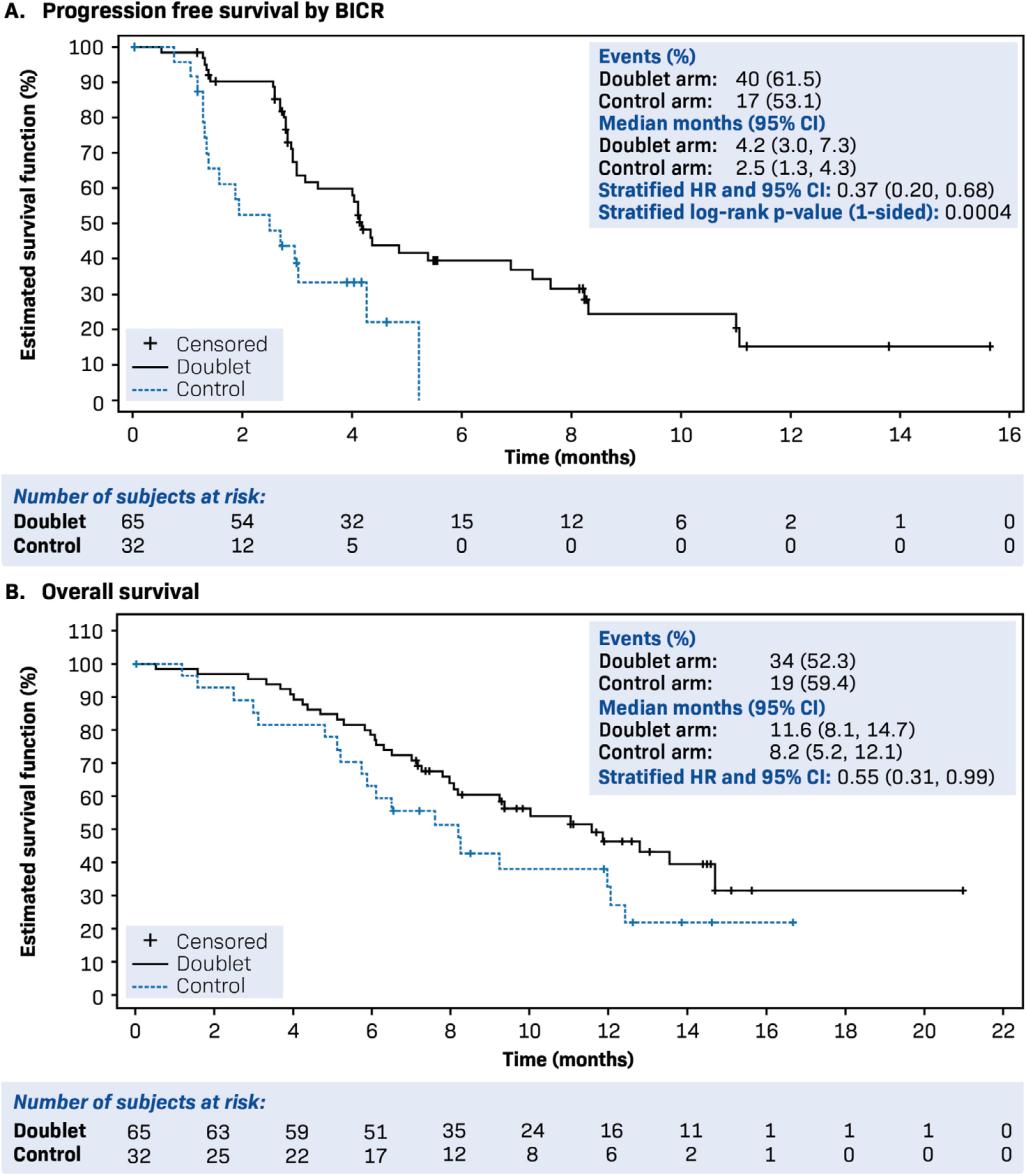
Progression free survival (A) and overall survival (B) by BICR (FAS). BICR, blinded independent central review; CI, confidence interval; FAS, full analysis set; HR, hazard ratio.

The potential follow‐up duration for PFS as estimated by the reverse Kaplan–Meier method was 8.2 months for the Doublet arm and 3.9 months for the Control arm. The median for PFS, assessed by BICR, was longer in the Doublet arm (4.2 months; 95% CI: 3.0–7.3 months) compared to the Control arm (2.5 months; 95% CI: 1.3–4.3 months), with a stratified HR of 0.37 (95% CI: 0.20–0.68), indicating a 63% reduction in the risk of disease progression or death for the Doublet arm. The stratified log‐rank test showed a highly statistically significant difference between the two arms (*p* = 0.0004), favoring the Doublet arm (Figure 1A).

The multivariate analysis of PFS showed that the treatment group (Doublet vs. Control) had a significantly lower hazard ratio (HR = 0.28, 95% CI: 0.14–0.58, *p* = 0.0006), indicating a strong treatment effect favoring the Doublet group. Other significant factors associated with improved PFS include male sex (HR = 0.52, 95% CI: 0.27–0.98, *p* = 0.0443), complete resection of the primary tumor (HR = 0.38, 95% CI: 0.19–0.73, *p* = 0.0038), lower baseline CRP (HR = 0.34, 95% CI: 0.15–0.78, *p* = 0.0106), and lower baseline CA 19‐9 (HR = 0.45, 95% CI: 0.21–0.94, *p* = 0.0326) (Table S1).

**FIGURE 2:**
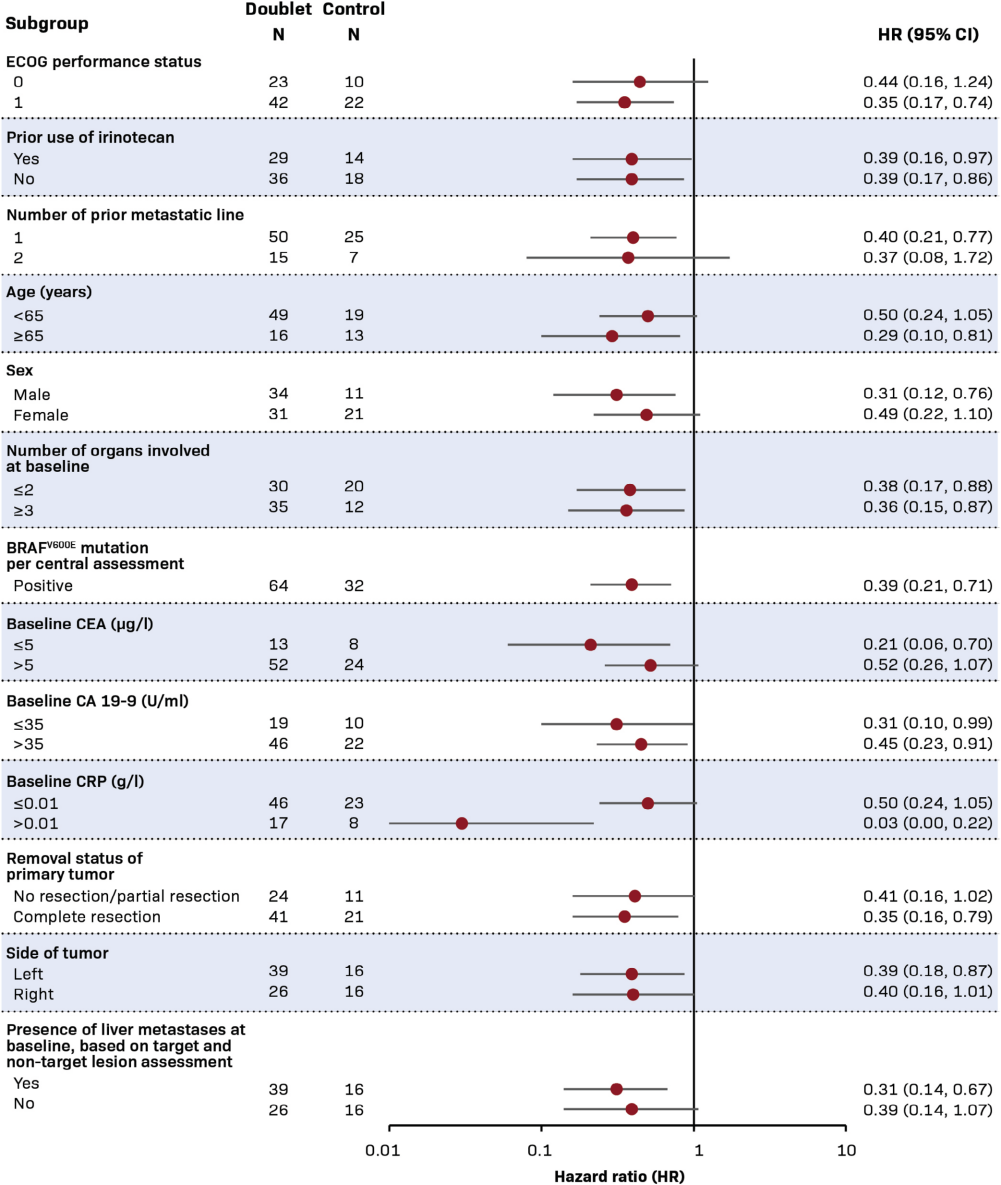
Progression‐free survival by BICR in subgroups (FAS). BICR, blinded independent central review; BRAF^V600E^, B‐Rapidly accelerating fibrosarcoma (BRAF) with a mutation of the BRAF gene in which valine (V) is substituted by glutamic acid (E) at amino acid 600; CA 19‐9, cancer antigen 19‐9; CEA, carcinoembryonic antigen; CI, confidence interval; CRP, C‐reactive protein; ECOG, Eastern Cooperative Oncology Group; FAS, full analysis set; HR, hazard ratio; *N*, number of patients; U, units.

All PFS subgroup analyses demonstrated HRs in favor of the Doublet arm. HR estimates are notably homogeneous in the different subgroups investigated. In subgroups with at least 10 patients in each arm, HR estimates range from 0.29 to 0.52 (Figure 2).

Per investigator assessment, PFS estimates were comparable to those determined by the BICR, with median PFS of 4.2 months (95% CI: 4.0–5.5) for the Doublet group and 2.5 months (95% CI: 1.3–3.9) for the Control group, resulting in a HR of 0.28 (95% CI: 0.16–0.50; *p* < 0.0001) (Figure S3).

##### 3.2.2.2 Confirmed Responses

As assessed by BICR, in the Randomized phase (Doublet, *N* = 65), two patients (3.1%, 95% CI: 0.4–10.7) had a CR, 14 patients (21.5%, 95% CI: 12.3–33.5) a PR, and 33 patients (50.8%, 95% CI: 38.1–63.4) achieved stable disease, with an ORR of 24.6% (95% CI: 14.8–36.9) and a DCR of 75.4% (95% CI: 63.1–85.2) (Table 2). In the Control arm (*N* = 32), assessed by BICR, the ORR was 6.3% (95% CI: 0.8–20.8) and DCR was 28.1% (95% CI: 13.7–46.7). Median DOR was 8.2 months in the randomized Doublet arm, and not estimable in the Control arm. The median TTR in the Randomized phase was 1.4 months for Doublet and 1.5 months for Control.

ORRs as assessed by the investigator were comparable across both the Doublet arm (26.2% [95% CI: 16.0–38.5]) and the Control arm (6.3% [95% CI: 0.8–20.8]) to those determined by the BICR.

##### 3.2.2.3 Overall Survival

The median OS for the Doublet arm was 11.6 months (95% CI: 8.1–14.7), compared to 8.2 months (95% CI: 5.2–12.1) in the Control arm. The stratified HR for overall survival was 0.55 (95% CI: 0.31–0.99), indicating a 45% reduction in the risk of death in the Doublet arm compared to the Control arm (Figure 1B). With a median follow‐up time of 12.6 months for both arms, as estimated by the reverse Kaplan–Meier method, the Doublet arm consistently showed improved survival over the Control arm throughout the study duration. In the Doublet arm, 34 deaths were reported (52.3% of patients), and 27 patients (41.5%) are still in follow‐up for survival. In the Control arm, 19 deaths were reported (59.4% of patients) and eight patients (25.0%) are still in follow‐up for survival.

##### 3.2.2.4 Post‐Progression Therapy

Overall, 56.9% of the Doublet arm (*n* = 37) and 65.6% of the Control arm (*n* = 21) received ≥ 1 subsequent systemic anticancer therapy. In the Doublet arm, 27.7% of patients received, as first subsequent systemic therapy, a combination of cytotoxic chemotherapy plus targeted therapy. In the Control arm, 34.4% of patients received, as first subsequent systemic therapy, a targeted therapy. Immunotherapy, alone or in combination, was provided as first subsequent therapy in 15.4% of the Doublet arm (*n* = 10) and 12.5% of the Control arm (*n* = 4).

#### 3.2.3 Safety and Tolerability

In the Randomized phase, the median duration of exposure was 18.9 weeks (range 1–68 weeks) for the Doublet arm and 8.0 weeks (range 2–22.6 weeks) for the Control arm. All patients in the SAF (*N* = 92) experienced at least one treatment‐emergent adverse event (TEAE) (Table 3). The estimated median time to onset of first TEAEs was 3.6 times longer in patients with events receiving the Doublet, compared to those receiving Control treatment (Doublet: 1.8 months [95% CI: 0.8–2.8]; Control: 0.5 month [95% CI: 0.3–1.4]). TEAEs of any grade (except COVID‐19) reported in either arm by more than 25% of patients were anemia (30.8% in the Doublet arm and 37.0% in the Control arm), vomiting (26.2% and 33.3%), and rash (24.6% and 29.6%, respectively). For other TEAEs, the safety profiles of each regimen differed. TEAEs with an absolute difference between groups of at least 10% included neutrophil/white blood cell decreased, raised aminotransferases, fatigue, and gastrointestinal disorders in the Control group than the Doublet group, whereas melanocytic naevus, myalgia, insomnia, and proteinuria were reported more frequently in the Doublet group (Table S2).

**TABLE 3:** Treatment‐emergent adverse events by SOC and PT in the Randomized phase (SAF).

*n* (%)	Randomized phase	
	Doublet (*N* = 65)	Control (*N* = 27)
**TEAEs**	**65 (100)**	**27 (100)**
**TEAEs (≥ grade 3)** Grade 3 Grade 4	**31 (47.7)**26 (40.0) 3 (4.6)	**14 (51.9)** 8 (29.6) 5 (18.5)
**Serious TEAEs**	**20 (30.8)**	**5 (18.5)**
**Serious TEAEs (≥ grade 3)**	**17 (26.2)**	**5 (18.5)**
**Serious TEAEs (≥ grade 4)**	**4 (6.2)**	**2 (7.4)**
**TEAE (grade 5)** Death Pneumonia Septic shock	**2 (3.1)** 1 (1.5) 1 (1.5) 0	**1 (3.7)** 0 0 1 (3.7)
**Most frequent TEAEs (except COVID‐19) > 20%** Anaemia Vomiting Rash Weight loss Hypoalbuminemia Melanocytic nevus	20 (30.8) 17 (26.2) 16 (24.6) 15 (23.1) 14 (21.5) 14 (21.5)	10 (37.0) 9 (33.3) 8 (29.6) 5 (18.5) 6 (22.2) —
**TEAEs leading to discontinuation of all study drug (any grade)**	**6 (9.2)**	**4 (14.8)**
**TEAEs leading to discontinuation of any study drug (≥ grade 3)**	**4 (6.2)**	**5 (18.5)**
**TEAEs leading to study intervention interruption (any drug)**	**35 (53.8)**	**12 (44.4)**
**TEAEs requiring additional therapies**	**56 (86.2)**	**26 (96.3)**
**Treatment‐related TEAE**	**62 (95.4)**	**27 (100)**
**Treatment‐related TEAEs (≥ grade 3)**	**16 (24.6)**	**12 (44.4)**
**Treatment‐related TEAEs (≥ grade 4)**	**3 (4.6)**	**6 (22.2)**
**Treatment‐related serious TEAEs**	**3 (4.6)**	**4 (14.8)**
**Treatment‐related serious TEAEs (≥ grade 3)**	**3 (4.6)**	**4 (14.8)**
**Treatment‐related serious TEAEs (≥ grade 4)**	**2 (3.1)**	**2 (7.4)**
**Most frequent treatment‐related TEAEs (PT > 20% in any treatment arm)** **Skin and subcutaneous tissue disorders** Rash **Investigations** Alanine aminotransferase increased Aspartate aminotransferase increased Neutrophil counts decreased White blood cell counts decreased **Gastrointestinal disorders** Vomiting Nausea Diarrhoea **Metabolism and nutrition disorders** Decreased appetite **Musculoskeletal and connective tissue disorders** **General disorders and administration site conditions** **Neoplasms benign, malignant, and unspecified (including cysts and polyps)** Melanocytic nevus **Blood and lymphatic system disorders** Anaemia	**34 (52.3)** 15 (23.1) **29 (44.6)** 8 (12.3) 7 (10.8) 4 (6.2) 2 (3.1) **23 (35.4)** 12 (18.5) 9 (13.8) 5 (7.7) **20 (30.8)** 8 (12.3) **20 (30.8)** **18 (27.7)** **16 (24.6)** 14 (21.5) **7 (10.8)** 6 (9.2)	**16 (59.3)** 8 (29.6) **19 (70.4)** 10 (37.0) 8 (29.6) 12 (44.4) 13 (48.1) **24 (88.9)** 9 (33.3) 9 (33.3) 9 (33.3) **11 (40.7)** 6 (22.2) 1 (3.7) **8 (29.6)** **0 (00.0)** 0 (00.0) **12 (44.4)** 9 (33.3)

*Note:* Bold numbers correspond to the main category; numbers in regular font correspond to the subcategories.

Abbreviations: ECOG, Eastern Cooperative Oncology Group; FAS, full analysis set; *n*, number of patients with the characteristic; *N*, number of patients; PS, performance status; SD, standard deviation; SLI, safety lead‐in.

Similarly, while comparable proportions of patients in each arm had a Grade ≥ 3 TEAE (47.7% in the Doublet arm and 51.9% in the Control arm), the distribution of Grade ≥ 3 TEAEs differed between groups. A lower incidence was observed in the Doublet arm as compared to the Control arm for TEAEs regardless of study drug relationship reported under the SOCs of investigations (18.5% vs. 29.6%), gastrointestinal disorders (13.8% vs. 25.9%), and blood and lymphatic system disorders (0% vs. 11.1%) (Table S2). The incidence of Grade 4 TEAEs was lower in the Doublet arm than in the Control arm (4.6% vs. 18.5%).

Almost all TEAEs were considered related to study intervention (Table 3). The incidence of related Grade ≥ 3 and Grade ≥ 4 TEAEs was notably lower in the Doublet arm (24.6% vs. 44.4% in the Control group for Grade ≥ 3 and 4.6% vs. 22.2% for Grade ≥ 4). The most frequent treatment‐related TEAEs included skin and subcutaneous tissue disorders, with rash being more prevalent in the Doublet arm (52.3%) compared to the Control arm (59.3%).

A higher percentage of patients in the Doublet experienced SAEs (30.8% vs. 18.5%) with few of them related to study treatment (4.6% vs. 14.8%). A lower percentage of patients in the Doublet arm compared with the Control arm experienced AEs leading to discontinuation of all study drugs (9.2% vs. 14.8%), AEs requiring dose reduction of any study drug (6.2% vs. 25.9%), and AEs requiring additional therapy (86.2% vs. 96.3%). The incidence of TEAEs leading to on‐treatment deaths was similar in the two treatment arms (3.1% and 3.7% for the Doublet and Control arms, respectively).

### 3.3 Quality of Life

At baseline, there was no significant difference in mean questionnaire scores between the Doublet and Control arms. However, when descriptively comparing changes from baseline over the first three cycles of treatment, the Doublet arm exhibited improvements across most dimensions of the questionnaires (data not shown). In contrast, the Control arm primarily experienced deteriorations, as assessed by the EORTC QLQ‐C30, EQ‐5D‐5L (including both EQ‐5D and EQ visual analog scale [VAS]), and FACT‐C measures (Figure S4). The EORTC QLQ‐C30 results indicated marked advantages for the Doublet arm regarding global health status, as well as physical, role, cognitive, and emotional functioning, with improvements noted across all symptoms except for sleep and constipation (data not shown).

The Doublet demonstrated superior results compared to the Control on repeated measurement analysis, with an adjusted mean difference of 15.71 (standard error [SE] 4.66, *p* = 0.0009) in QLQ‐C30 global health status score (Figure S5A). Additionally, there was an estimated 86% reduction in the risk of a definitive 10% deterioration in the EORTC QLQ‐C30 global health status score for the Doublet arm relative to the Control arm (stratified HR 0.14, 95% CI: 0.06–0.33). The estimated median time to a definitive 10% deterioration was significantly longer in the Doublet arm at 8.3 months (95% CI: 7.3, not evaluable [NE]) compared to 2.5 months (95% CI: 1.6, NE) in the Control arm (Figure S4A).

EQ‐5D‐5L EQ VAS results indicated no significant difference between the Doublet and Control arms, with a difference (SE) in adjusted means of 9.86 (5.14), *p* = 0.0558 (data not shown).

The FACT‐C Total score results demonstrated that the Doublet arm outperformed the Control arm, with an adjusted mean difference of 17.58 (SE 4.97, *p* = 0.0005) (Figure S5B). Furthermore, there was an estimated 75% reduction in the risk of a definitive 10% deterioration in the FACT‐C Total score for the Doublet arm compared to the Control arm (stratified HR 0.25, 95% CI: 0.13–0.51). The estimated median time to definitive 10% deterioration was significantly longer in the Doublet arm at 9.4 months (95% CI: 5.6, NE) compared to 2.5 months (95% CI: 1.4, 5.9) in the Control arm (Figure S4C).

The PGIC results indicated that a majority of patients in the Doublet arm reported feeling much or very much improved during their treatment cycles, with percentages ranging from 59.0% to 92.9% over the first 12 cycles. In contrast, only 17.6% of Control‐treated patients reported similar improvement by Cycle 2, which increased to 27.3% by Cycle 3 (data not shown).

## 4 Discussion

Results from the Phase II NAUTICAL CRC study (cut‐off: December 19, 2023) demonstrate the clinical benefit of the combination treatment of encorafenib and cetuximab, referred to as the Doublet regimen, compared to the standard of care in a cohort of Chinese patients with BRAF^V600E^ mutant mCRC.

The Randomized phase II started after an independent Data Monitoring Committee confirmed that the Doublet was well tolerated, with especially no toxicity of grade 3 or more across the first cycle of therapy in the SLI population.

A central review was performed to limit the potential for bias in assessing response in the Randomized phase, the study involved a 2:1 ratio randomization of participants, respectively, to the Doublet arm and the Control arm. As this is a bridging study, the unequal randomization in a 2:1 manner allowed a more efficient study, as it minimized the number of control participants exposed to the control treatment while both maintaining the benefit/risk as well as ensuring the number of prespecified events occur to adequately power this study [23].

Demographically, patients in the Randomized phase had a mean age of 53.87 years, and 70% were younger than 65 years. Importantly, the overall enrolled population had a high prevalence of elevated biomarkers such as CEA, CA 19‐9, and CRP, highlighting their advanced disease status. The population in the NAUTICAL study is consistent with the targeted population.

The median duration of exposure to study treatment was substantially longer in the Doublet arm versus the Control arm, reflecting the higher PFS in the Doublet arm as PD was an indication for treatment discontinuation, and the higher proportion of participants in the Control arm who discontinued treatment due to TEAE.

The study's primary efficacy endpoint, PFS, demonstrated that the Doublet regimen provided a significant benefit compared to the Control arm. In the Chinese cohort, the median PFS in the Doublet arm was 4.2 months, compared to 2.5 months in the Control arm, with an HR of 0.37, reflecting a 63% reduction in the risk of progression or death (*p* = 0.0004). This finding is consistent with previous studies that explored the efficacy of targeted therapies in BRAF‐mutated mCRC in a non‐Chinese cohort. For instance, the BEACON CRC study also demonstrated that the combination of encorafenib and cetuximab improved PFS, with a reported median PFS of 4.3 months compared to 1.5 months in the Control arm [15, 18]. Our study reaffirmed the benefit of BRAF‐targeted therapies in improving PFS outcomes, consistent with the broader research context.

The multivariate Cox analysis, after adjusting for prespecified covariates, showed consistent results with the primary PFS analysis, indicating that the HR for PFS is in favor of the Doublet regimen (HR = 0.28, *p* = 0.0006). The benefit of the Doublet regimen was seen across all investigated subgroups, and predictive factors such as sex, removal status of primary tumor, baseline CRP, and CA 19‐9 levels were strongly associated with improved outcomes for males, complete tumor resection, higher CRP and lower CA 19‐9 at baseline. These findings align with known predictive indicators in mCRC. Higher CRP and lower CA 19‐9 levels have been associated with improved outcomes, as these biomarkers are indicative of systemic inflammation and tumor burden, respectively [24]. The sex differences observed in the study are interesting, as some studies suggest that sex‐specific differences are important in the management of mCRC worldwide, although the reasons for this are not fully understood [25, 26, 27].

In terms of confirmed responses, the Doublet arm achieved a median DOR of 8.2 months, whereas the DOR in the Control arm was not estimable. Stable disease was also significantly higher in the Doublet arm (50.8%) than in the Control arm (28.1%), suggesting that the combination therapy was more successful in disease stabilization. The OS analysis further confirmed the superiority of the Doublet regimen, with a median OS of 11.6 months vs. 8.2 months in the Control arm, and a stratified HR of 0.55, indicating a 45% reduction in the risk of death. This is consistent with results from the BEACON CRC study, where the median OS was 9.3 months (95% CI: 8.0–11.3) in the Doublet arm (encorafenib + cetuximab) compared to 5.9 months (95% CI: 5.1–7.1) in the Control arm [15, 18]. These results collectively reinforce the notion that targeting the BRAF^V600E^ mutation with encorafenib and cetuximab is a viable strategy for improving survival outcomes in this patient population.

QoL assessments favored the Doublet regimen, revealing that patients treated with this approach exhibited improvements or stability across most dimensions of QoL as measured by the EORTC QLQ‐C30, EQ‐5D‐5L, and FACT‐C questionnaires. Specifically, the Doublet arm demonstrated better global health status, along with enhanced physical and emotional functioning, and fewer symptoms compared to the Control arm. This positive impact on QoL, coupled with the clinical benefits observed, further underscores the potential of the Doublet regimen as a superior treatment option for mCRC. These findings are consistent with prior research indicating that targeted therapies can enhance QoL compared to traditional chemotherapy, particularly due to a reduction in severe side effects such as fatigue and nausea [28].

The safety profile of the Doublet regimen was generally manageable and appeared to be more favorable than the Control arm, as evidenced by less frequent discontinuation due to TEAEs in the Doublet arm than in the Control arm, fewer dose reductions of any study drug, and fewer Grade ≥ 3 and Grade 4 TEAEs in the Doublet arm compared to the Control. This suggests that while both treatments are associated with TEAEs, the combination therapy appears less likely to result in severe complications. A higher percentage of patients in the Doublet arm experienced SAEs compared to those in the Control arm (30.8% vs. 18.5%), with a smaller proportion of these SAEs being related to the study treatment (4.6% vs. 14.8%). The incidence of adverse events leading to death was similar between the two treatment arms (3.1% in the Doublet arm and 3.7% in the Control arm).

The safety and tolerability profile of doublet treatment is consistent with the known profiles of the component agents. Results are consistent with the results of the BEACON CRC study [3, 15, 19, 20].

Study limitations that may affect the generalizability and robustness of its findings include the relatively short follow‐up duration, which constrains long‐term assessment of OS, and the restricted sample size for the subgroup analyses that hampers their interpretation, although the direction of the HR was consistent across the subgroup analyses. High withdrawal rates from the Control arm after randomization reflect the open‐label design, which may also have introduced risks of bias. Fully informed consent discussion (study randomization procedure, reassurance on the control arm as a well‐known and tested regimen, added benefit of high level of monitoring and care) before randomization is a critical step for Chinese patient acceptance to control treatment. Nevertheless, the consistency of the results with the BEACON study supports the validity of our findings.

## 5 Conclusions

In conclusion, the combination of encorafenib and cetuximab is the first chemotherapy‐free treatment regimen that provides a significant clinical advantage over the standard of care in Chinese patients with mCRC previously treated. This advantage is evident through improvements in all efficacy endpoints, including PFS and OS, along with a manageable safety profile. Additionally, the Doublet regimen offers QoL benefits, which are important in the treatment of patients with advanced cancer. Given these results, Doublet therapy should be regarded as a new option in the therapeutic landscape for Chinese patients with previously treated BRAFV600E mutant mCRC.

## Author Contributions

Shen Lin, Klauck Isabelle, and Vedovato Jean‐Claude: conceptualization. Shen Lin, Klauck Isabelle, Vedovato Jean‐Claude, and Groc Mélanie: data curation. Wang Xicheng, Deng Yanhong, Zhang Yanqiao, Liu Tianshu, Yuan Xianglin, Yang Jianwei, Zhang Tao, Zang Aimin, Liu Yu, Huang Li, Ye Feng, Zong Hong, Ba Yi, Guo Angela, Li Jian, and Shen Lin: investigation. Shen Lin, Klauck Isabelle, Vedovato Jean‐Claude, and Groc Mélanie: methodology. Klauck Isabelle and Vedovato Jean‐Claude: project administration. Wang Xicheng, Deng Yanhong, Zhang Yanqiao, Liu Tianshu, Yuan Xianglin, Yang Jianwei, Zhang Tao, Zang Aimin, Liu Yu, Huang Li, Ye Feng, Zong Hong, Ba Yi, Klauck Isabelle, Vedovato Jean‐Claude, Groc Mélanie, Guo Angela, Li Jian, and Shen Lin: writing – review and editing.

## Funding

Pierre Fabre funded this research (Study identifier: W00090GE202) and was involved in all stages of study conduct, including analysis of the data. Pierre Fabre also took charge of all costs associated with the development and publication of this manuscript.

## Ethics Statement

The study was approved by the institutional review board or independent ethics committee at each center and was conducted in accordance with the requirements of the regulatory authorities and with the provisions of the Declaration of Helsinki and the Good Clinical Practice guidelines of the International Council on Harmonization. The ethical approval numbers for each site are as follows: Beijing Cancer Hospital: 2021YW47; The Sixth Affiliated Hospital, Sun Yat‐sen University: 2021ZSLYEC‐303; Peking University Shenzhen Hospital: Peking University Shenzhen Hospital Ethical Review [2021] No.: (045); Tongji Hospital, Tongji Medical College Huazhong University of Science and Technology: [2021] Ethical Review No. (133); The First Hospital of Jilin University: 21Y161‐002; The First Affiliated Hospital, Zhejiang University School of Medicine: 2021 Ethical Review No. (482); Sir Run Run Shaw Hospital, Zhejiang University School of Medicine: Drug Clinical Trial 20210803‐6; Harbin Medical University Cancer Hospital: 2021‐208; Peking Union Medical College Hospital, Chinese Academy of Medical Sciences: KS2022587; Xinhua Hospital Affiliated to Shanghai Jiao Tong University School of Medicine: XHEC‐A‐2021‐023‐1; Liaoning Cancer Hospital & Institute: 20210904; Fujian Cancer Hospital: 2021‐123‐01; Henan Cancer Hospital: 2021‐195‐003; The First People's Hospital of Changzhou: (2021) Drug No. 023; Shanghai East Hospital, Tongji University: [2021] Preliminary review No. (068); Union Hospital, Tongji Medical College of Huazhong University of Science and Technology: [2021] Ethical Review No. (0216); Affiliated Hospital of Hebei University: HDFY‐LL‐2021‐119; The Second Affiliated Hospital of Nanchang University: Drug Clinical Review [2021] No. (56); The First Affiliated Hospital of Nanchang University: 2021 Clinical Ethics Review No. 159; The Second People's Hospital of Neijiang: Nei Er Yi 2021‐016‐001; Xiangya Hospital Central South University: Ethical Review GCP No. (Expedited 202111538); First Affiliated Hospital of Gannan Medical University: (2021) GYLS No. 062; The First Hospital of China Medical University: 2021YL107; Cancer Hospital of Shantou University Medical College: 2021024; The First Affiliated Hospital of Xiamen University: [2021] Drug Ethical Review No. (106); Shaanxi Provincial People's Hospital: (2021) Ethical Review No.: (Y015); The First Affiliated Hospital of Zhengzhou University: L2021‐Y100‐002; Zhongshan Hospital Fudan University: 2021‐169; Tianjin Medical University Cancer Institute and Hospital: E20210546; Cancer Center of Guangzhou Medical University: Medical Ethics First Review [2021] No. 22; Huashan Hospital Fudan University: 2021 Clinical Approval No. (764); Wuhan Zhongnan Hospital: Drug Ethical [2022009]; The First Affiliated Hospital of Jinzhou Medical University: 202201; The First People's Hospital of Foshan: Drug Ethical Review [2022] No. 6.

## Conflicts of Interest

Shen Lin: Consulting or Advisory Role for AstraZeneca; Boehringer Ingelheim; MSD; SERVIER; Transcenta Holding Limited and Research Funding—BeiGene (Institution). Klauck Isabelle, Groc Mélanie, Vedovato Jean‐Claude, and Guo Angela are employed by Pierre Fabre. The other authors declare no financial and nonfinancial relationships and activities and no conflicts of interest.

## Supporting information


**Table S1:** Progression‐free survival based on BICR—Multivariate analyses (FAS).
**Table S2:** Treatment‐emergent adverse events (TEAE) by SOC and PT in the Randomized phase: Any TEAE reported by at least 15% of participants in either group and Grade ≥ 3 TEAEs reported by at least 2 participants in either group (SAF).
**Figure S1:** Study design.
**Figure S2:** Patient disposition (CONSORT flow chart).
**Figure S3:** Progression free survival by investigator assessment (FAS).
**Figure S4:** Time to definitive deterioration in Patient‐Reported Outcomes for EORTC QLQ‐C30 (A), EQ‐5D‐5L (B), FACT‐C (C) in (FAS).
**Figure S5:** (A) EORTC QLQ‐C30 changes from baseline in Global Health Status. (B) FACT‐C Total score—Changes from Baseline.

## Data Availability

Pierre Fabre makes available the anonymized individual patient data and associated documents from interventional clinical studies which evaluate medicines, upon approval of proposals submitted to isabelle.klauck@pierre-fabre.com. To access data for other types of Pierre Fabre sponsored research, for study documents without patient‐level data and for clinical studies not listed, please submit an enquiry via the website.
