# Supplementary material for: A Randomized Trial of Encorafenib and Cetuximab Versus Irinotecan/Cetuximab or FOLFIRI/Cetuximab in Chinese Patients With BRAFV600E Mutant Metastatic Colorectal Cancer: The NAUTICAL Study
Source: Cancer Med. 2026 Mar 19;15(3):e71697. doi: 10.1002/cam4.71697 (PMC13093443; doi:10.1002/cam4.71697)
Supplement: Supplementary file 1 — Table S1: Progression‐free survival based on BICR—Multivariate analyses (FAS). Table S2: Treatment‐emergent adverse events (TEAE) by SOC and PT in the Randomized phase: Any TEAE reported by at least 15% of participants in either group and Grade ≥ 3 TEAEs reported by at least 2 participants in either group (SAF). Figure S1: Study design. Figure S2: Patient disposition (CONSORT flow chart). Figure S3: Progression free survival by investigator assessment (FAS). Figure S4: Time to definitive deterioration in Patient‐Reported Outcomes for EORTC QLQ‐C30 (A), EQ‐5D‐5L (B), FACT‐C (C) in (FAS). Figure S5: (A) EORTC QLQ‐C30 changes from baseline in Global Health Status. (B) FACT‐C Total score—Changes from Baseline. [file CAM4-15-e71697-s001.docx]

**Supplementary Tables**

**Supplementary Table 1:** Progression-Free Survival based on BICR – Multivariate analyses (FAS)

|  | **Hazard ratio** | **95% CI** | **p-value**  **(two-sided)** |
| --- | --- | --- | --- |
| **Treatment group** (Doublet vs. Control) | 0.28 | 0.14–0.58 | 0.0006 |
| **Sex** (Male vs. Female) | 0.52 | 0.27–0.98 | 0.0443 |
| **Age** (<65 vs. ≥65 years) | 0.62 | 0.30–1.27 | 0.1889 |
| **Removal status of primary tumor**  (Complete resection vs. Partial resection/Unresected) | 0.38 | 0.19–0.73 | 0.0038 |
| **CRP baseline level** (≤0.01 vs. >0.01 g/L) | 0.34 | 0.15–0.78 | 0.0106 |
| **Baseline CEA** (≤5 vs. >5 μg/L) | 0.81 | 0.37–1.80 | 0.6122 |
| **Baseline CA 19-9** (≤35 vs. >35 U/mL) | 0.45 | 0.21–0.94 | 0.0326 |
| **Side of tumor** (left vs. right) | 1.49 | 0.72–3.08 | 0.2772 |
| **Number of organs involved based on target and non-target lesion assessment^†^** (≤2 vs. 3+) | 1.10 | 0.53–2.25 | 0.8009 |
| **Presence of liver metastases at baseline, based on target and non-target lesion assessment** (Yes vs. No) | 1.75 | 0.83–3.70 | 0.1424 |
| **Number of prior metastatic line** (1 vs. 2) | 1.63 | 0.67–3.94 | 0.2809 |
| **Prior oxaliplatin** (Yes vs. No) | 1.05 | 0.39–2.84 | 0.9303 |

**^†^**Based on investigator assessment.

Multivariate Cox proportional hazard models are used to evaluate the effect of confounding variables on PFS with ECOG performance status (0 vs. 1) and prior use of irinotecan (yes vs. no) as stratification factors.

**Abbreviations:** BICR, blinded independent central review; CA 19-9, cancer antigen 19-9; CEA, carcinoembryonic antigen; CI, confidence interval; CRP, C-reactive protein; ECOG, Eastern Cooperative Oncology Group; FAS, full analysis set; PFS, progression free survival.

**Supplementary Table 2:** Treatment-emergent adverse events (TEAE) by SOC and PT in the Randomized phase: Any TEAE reported by at least 15% of participants in either group and Grade ≥3 TEAEs reported by at least 2 participants in either group (SAF)

|  | **Randomized phase** | | | |
| --- | --- | --- | --- | --- |
|  | **Doublet (N=65)** | | **Control (N=27)** | |
|  | All Grades n (%) | Grade ≥3 n (%) | All Grades n (%) | Grade ≥3 n (%) |
| **Any Treatment Emergent Adverse Event** | 65 (100) | 31 (47.7) | 27 (100) | 14 (51.9) |
|  |  |  |  |  |
| **Investigations** | 43 (66.2) | 12 (18.5) | 20 (74.1) | 8 (29.6) |
| Weight decreased | 15 (23.1) | 0 | 5 (18.5) | 0 |
| Aspartate aminotransferase increased | 13 (20.0) | 1 (1.5) | 9 (33.3) | 0 |
| Alanine aminotransferase increased | 12 (18.5) | 0 | 11 (40.7) | 0 |
| Lipase increased | 8 (12.3) | 3 (4.6) | 1 (3.7) | 0 |
| Blood alkaline phosphatase increased | 7 (10.8) | 2 (3.1) | 1 (3.7) | 0 |
| Neutrophil count decreased | 7 (10.8) | 2 (3.1) | 12 (44.4) | 6 (22.2) |
| White blood cell count decreased | 5 (7.7) | 0 | 13 (48.1) | 5 (18.5) |
| White blood cells urine positive | 1 (1.5) | 0 | 2 (7.4) | 0 |
| **Gastrointestinal disorders** | 38 (58.5) | 9 (13.8) | 24 (88.9) | 7 (25.9) |
| Vomiting | 17 (26.2) | 0 | 9 (33.3) | 0 |
| Nausea | 12 (18.5) | 0 | 9 (33.3) | 0 |
| Abdominal pain | 10 (15.4) | 2 (3.1) | 5 (18.5) | 0 |
| Diarrhoea | 7 (10.8) | 0 | 10 (37.0) | 2 (7.4) |
| Intestinal obstruction | 5 (7.7) | 5 (7.7) | 3 (11.1) | 2 (7.4) |
| Gastrointestinal perforation | 0 | 0 | 2 (7.4) | 2 (7.4) |
| **Skin and subcutaneous tissue disorders** | 38 (58.5) | 2 (3.1) | 17 (63.0) | 0 |
| Rash | 16 (24.6) | 1 (1.5) | 8 (29.6) | 0 |
| Dermatitis acneiform | 10 (15.4) | 0 | 4 (14.8) | 0 |
| **Infections and infestations** | 34 (52.3) | 4 (6.2) | 9 (33.3) | 1 (3.7) |
| COVID-19 | 25 (38.5) | 1 (1.5) | 6 (22.2) | 0 |
| Pneumonia | 4 (6.2) | 3 (4.6) | 0 | 0 |
| **Metabolism and nutrition disorders** | 33 (50.8) | 4 (6.2) | 13 (48.1) | 2 (7.4) |
| Hypoalbuminaemia | 14 (21.5) | 0 | 6 (22.2) | 0 |
| Decreased appetite | 11 (16.9) | 0 | 6 (22.2) | 0 |
| Hyponatraemia | 9 (13.8) | 2 (3.1) | 4 (14.8) | 1 (3.7) |
| Hypomagnesaemia | 5 (7.7) | 2 (3.1) | 0 | 0 |
| **General disorders and administration site conditions** | 28 (43.1) | 2 (3.1) | 9 (33.3) | 0 |
| Pyrexia | 10 (15.4) | 1 (1.5) | 2 (7.4) | 0 |
| Fatigue | 4 (6.2) | 0 | 6 (22.2) | 0 |
| **Musculoskeletal and connective tissue disorders** | 28 (43.1) | 3 (4.6) | 2 (7.4) | 0 |
| Myalgia | 12 (18.5) | 1 (1.5) | 0 | 0 |
| **Blood and lymphatic system disorders** | 21 (32.3) | 0 | 13 (48.1) | 3 (11.1) |
| Anaemia | 20 (30.8) | 0 | 10 (37.0) | 2 (7.4) |
| **Neoplasms benign, malignant and unspecified (incl cysts and polyps)** | 18 (27.7) | 0 | 0 | 0 |
| Melanocytic naevus | 14 (21.5) | 0 | 0 | 0 |
| **Nervous system disorders** | 17 (26.2) | 4 (6.2) | 6 (22.2) | 0 |
| Headache | 7 (10.8) | 2 (3.1) | 0 | 0 |
| **Renal and urinary disorders** | 16 (24.6) | 2 (3.1) | 3 (11.1) | 0 |
| Proteinuria | 10 (15.4) | 1 (1.5) | 0 | 0 |
| **Psychiatric disorders** | 12 (18.5) | 0 | 1 (3.7) | 0 |
| Insomnia | 11 (16.9) | 0 | 1 (3.7) | 0 |

**Abbreviations:** COVID-19, coronavirus disease 2019; N, number of patients; n, number of patients with the event; PT, preferred term; SAF, safety analysis set; SOC, System Order Class; TEAEs, treatment-emergent adverse events.

**Supplementary Figures**

**Supplementary Figure 1:** Study design


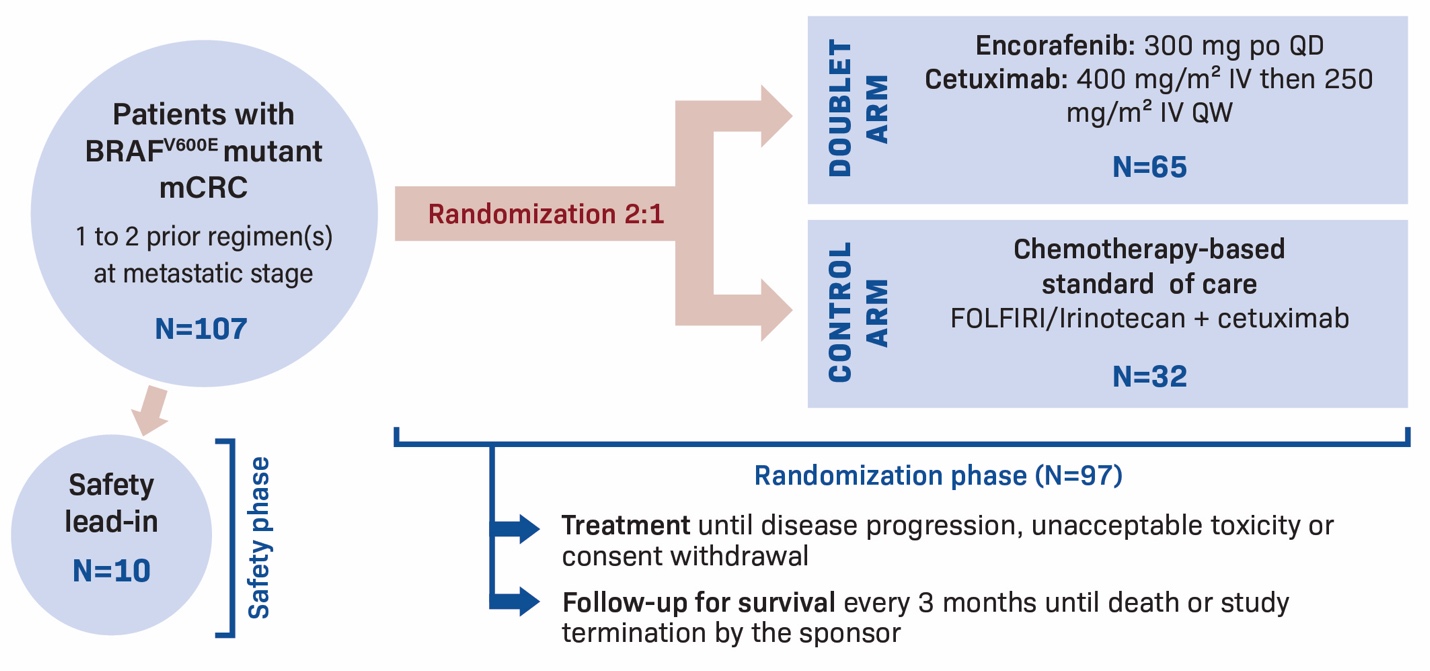


BRAF^V600E^, B-Rapidly accelerating fibrosarcoma (BRAF) with a mutation of the BRAF gene in which valine (V) is substituted by glutamic acid (E) at amino acid 600; IV, intravenous; mCRC, metastatic colorectal cancer; N, number of patients; po, per os (by mouth); QD, every day; QW, every week.

**Supplementary Figure 2:** Patient disposition (CONSORT flow chart)


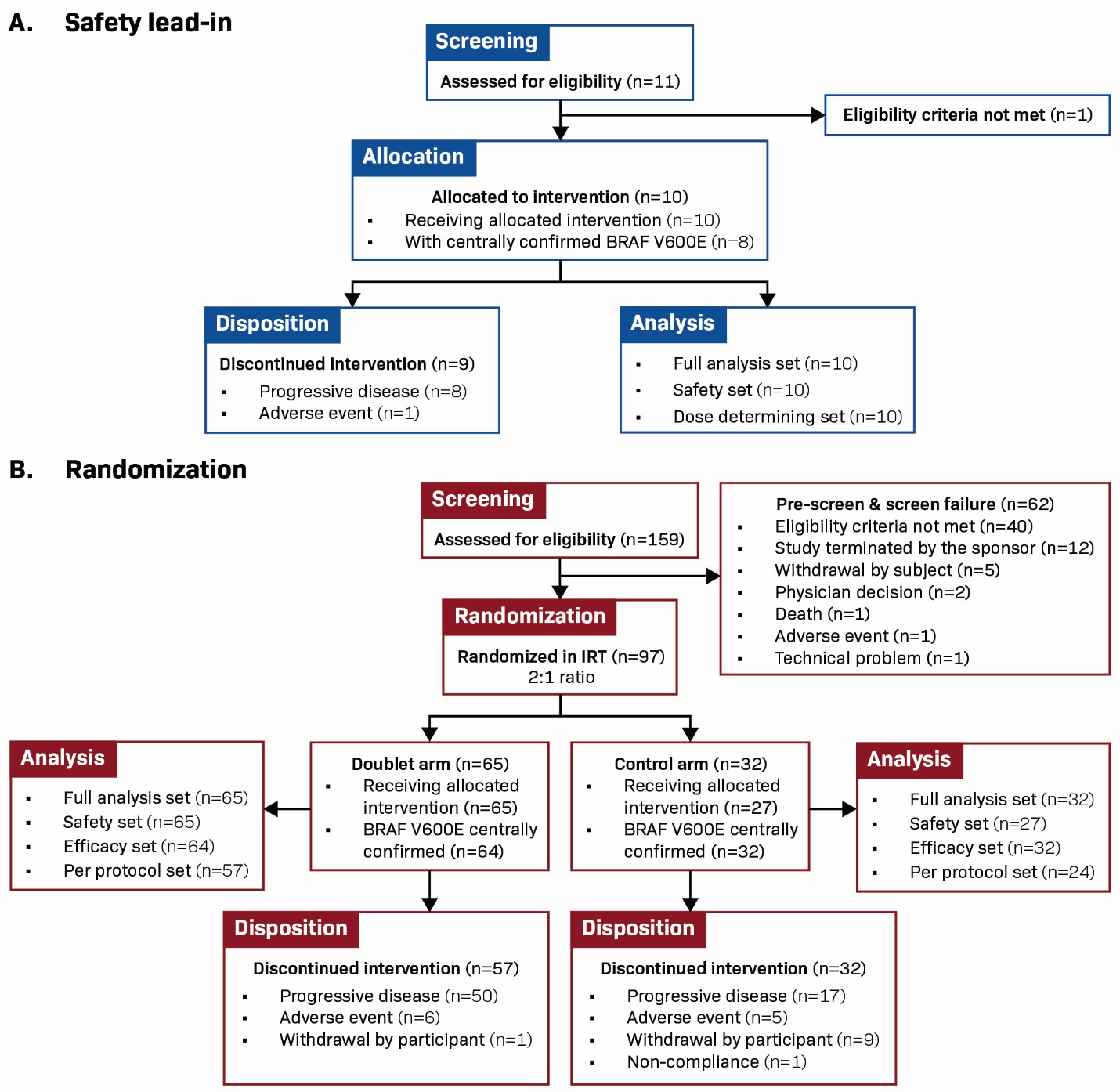


Five patients withdrew consent prior to randomization with no reason specified. Among 10 patients who withdrew consent after randomization, five were never treated with study drug (two of them refused to receive the control regimen), and five discontinued study drug during the treatment (two due to poor tolerability or AEs, surgery not allowed as per protocol for one, and unspecified for the other two).

AE, adverse event; BRAF^V600E^, B-Rapidly accelerating fibrosarcoma (BRAF) with a mutation of the BRAF gene in which valine (V) is substituted by glutamic acid (E) at amino acid 600; IRT, Interactive Response Technology; n, number of patients.

**Supplementary Figure 3:** Progression free survival by investigator assessment (FAS)


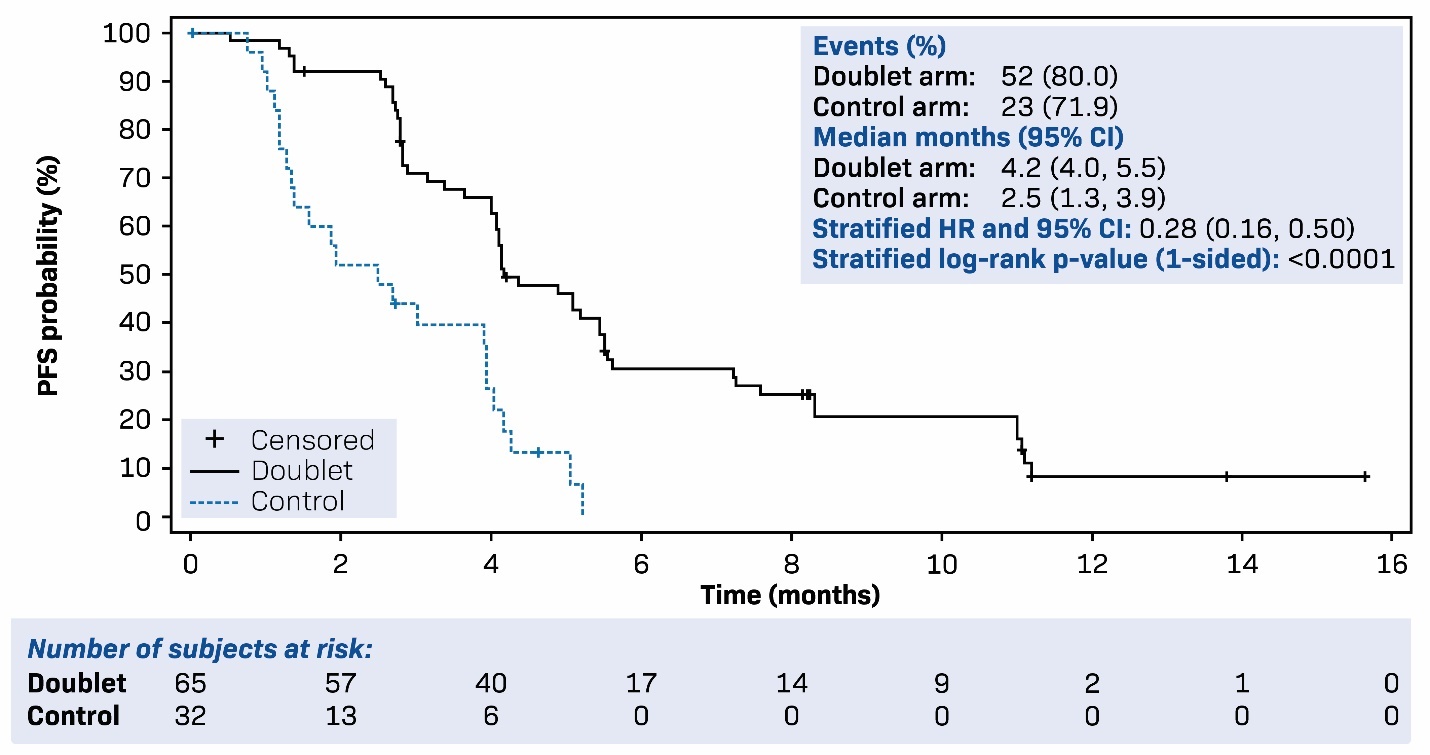


CI, confidence interval; FAS, full analysis set; HR, hazard ratio; PFS, progression free survival.

**Supplementary Figure 4:** Time to definitive deterioration in Patient-Reported Outcomes for EORTC QLQ-C30 **(A),** EQ-5D-5L **(B)**, FACT-C **(C)** in (FAS)


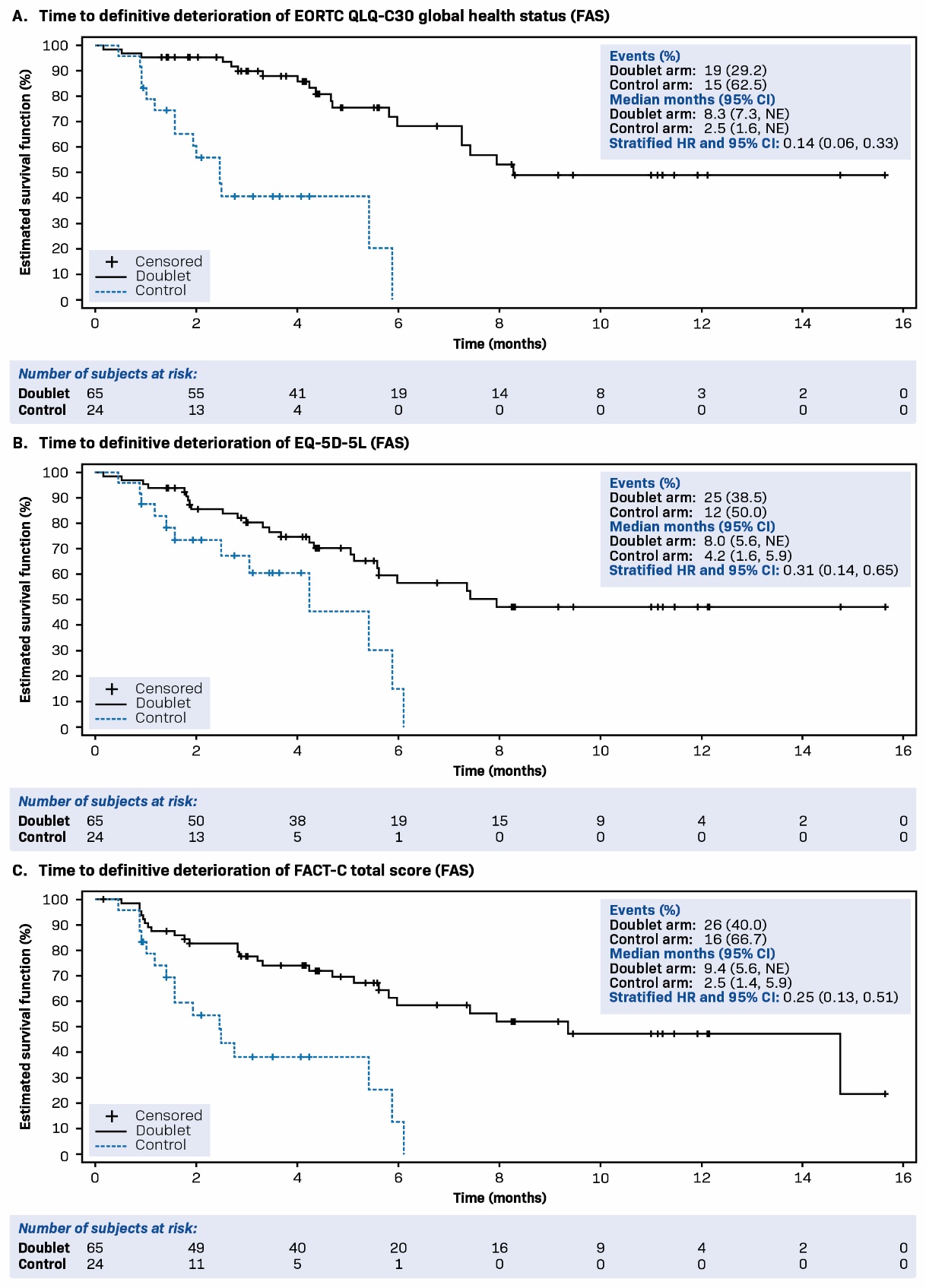


CI, confidence interval; EORTC QLQ-C30, European Organisation for Research and Treatment of Cancer Quality of Life Questionnaire-Core 30; EQ-5D-5L, EuroQoL 5-Dimension 5-Level; FACT-C, Functional Assessment of Cancer Therapy-Colon Cancer; FAS, full analysis set; HR, hazard ratio; NE, not evaluable; QoL, quality of life.

**Supplementary Figure 5: A)** EORTC QLQ-C30 changes from baseline in Global Health Status **B)** FACT-C Total score - Changes from Baseline

**
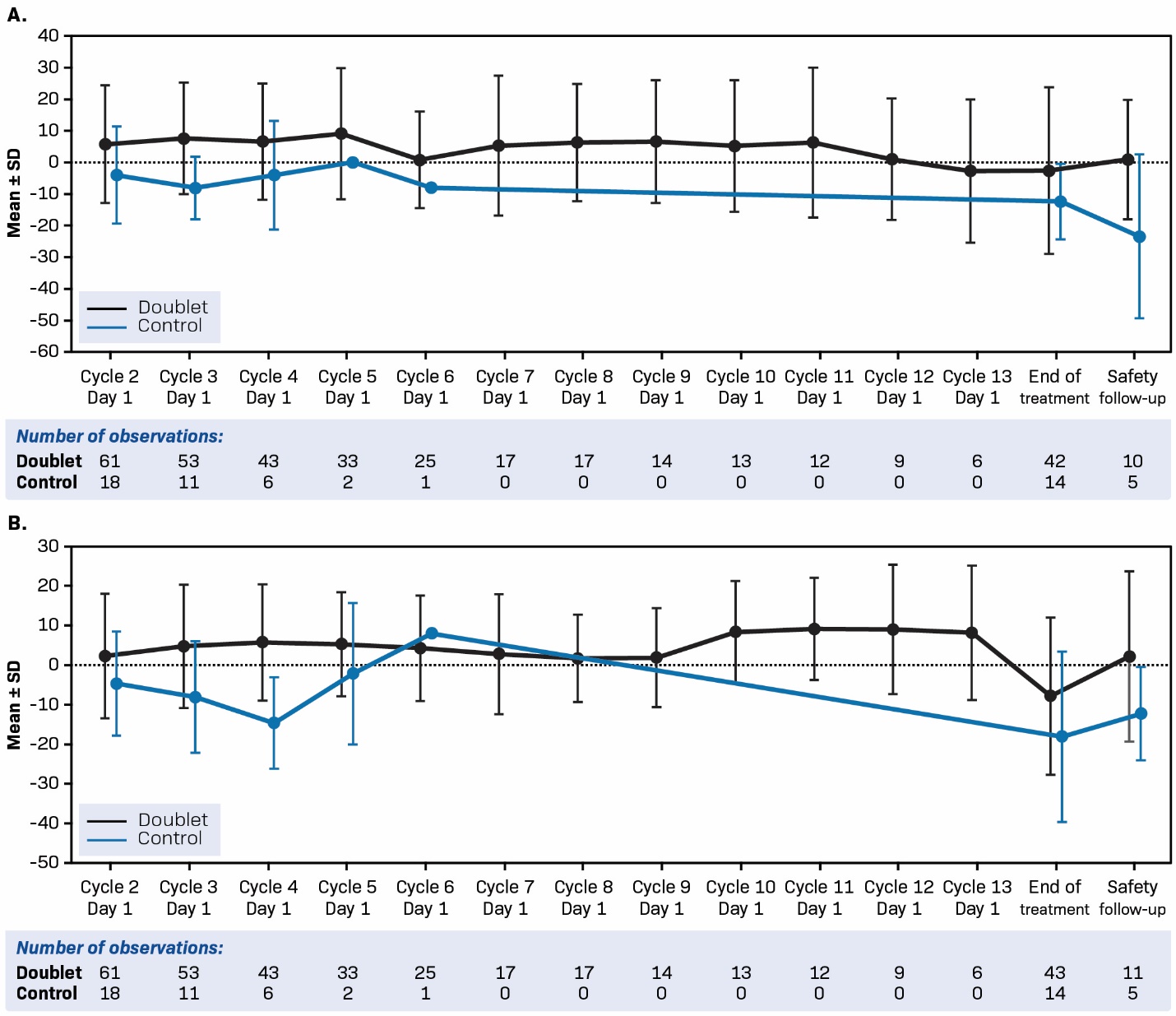
**

Note: End of treatment visit was performed at the time of study intervention discontinuation, as soon as possible and ≤14 days after the last dose of study intervention

Safety follow-up refers to the mean change over the entire follow-up period

EORTC QLQ-C30, European Organisation for Research and Treatment of Cancer Quality of Life Questionnaire-Core 30; FACT-C, Functional Assessment of Cancer Therapy-Colon Cancer; SD, standard deviation.

**Supplementary Information**

**Supplementary Methods**

**Study participants**

Chinese male and female patients were eligible for the NAUTICAL study if they were at least 18 years old at the time of providing informed consent. Patients were required to have documented histology or cytology-confirmed colorectal cancer (CRC) that was metastatic and unresectable at the time of study entry, with the presence of a BRAF^V600E^ mutation in tumor tissue determined by a prior local assay and confirmed by a central laboratory. Additionally, their disease must have progressed after one or two prior treatment lines in the metastatic setting. Patients were also required to be treatment-naïve to BRAF inhibitors, cetuximab, panitumumab, or other epidermal growth factor receptor (EGFR) inhibitors. They needed to have measurable disease according to Response Evaluation Criteria in Solid Tumors (RECIST) v1.1 criteria, an Eastern Cooperative Oncology Group (ECOG) performance status of 0 or 1, and adequate hematologic, hepatic, and renal function.

Patients were excluded from this study if they had a known contraindication to receiving cetuximab or irinotecan at the planned dose, a known history of Gilbert's syndrome, symptomatic brain metastasis, or leptomeningeal disease. Other exclusion criteria included a known history of acute or chronic pancreatitis, chronic inflammatory bowel disease, Crohn's disease, impaired cardiovascular function, or clinically significant cardiovascular diseases. Patients with uncontrolled severe hypertension, impaired gastrointestinal function or disease that might significantly alter the absorption of encorafenib were also excluded. Additionally, patients were excluded if they had a known history of a positive test for human immunodeficiency virus, active hepatitis B virus, hepatitis C virus, or any other severe active viral infection, such as severe acute respiratory syndrome coronavirus infection.

**Definition of Anti-tumor Activity Outcomes**

- **Dose-Limiting Toxicity (DLT) rates defined as:** Number of DLT-evaluable patients with DLTs in the DLT-evaluation period divided by the number of DLT-evaluable patients
- **Progression-Free Survival (PFS) defined as:** Time from the date of randomization to the earliest documented date of disease progression, or death due to any cause
- **Objective Response Rate (ORR) defined as:** Proportion of patients with a confirmed (resp. unconfirmed) best overall response of either complete response (CR) or partial response (PR)
- **Duration of Response (DOR) defined as:** Time from the date of the first documented response (CR or PR) to the earliest date of disease progression, or death due to any cause
- **Disease Control Rate (DCR) defined as:** Proportion of patients with a best overall response of either CR, PR or stable disease
- **Time to Response (TTR) defined as:** Time between date of randomization until first documented response of CR or PR
- **Overall Survival (OS) defined as:** Time between date of randomization until date of death due to any cause.
- Confirmed responses, including Best Overall Response (BOR), ORR, and DCR, were reported as the number and percentage of patients, along with a 95% Clopper-Pearson confidence interval (CI).

**Definition of Safety and Tolerability Outcomes**

Type and severity of adverse events (AEs) and serious AEs (SAEs), changes in physical examinations, vital signs, electrocardiogram (ECGs), clinical safety laboratory assessment values, dermatological examinations and performance status using the ECOG performance status scale

**Definition of Quality of Life Outcomes**

Change from baseline in the following questionnaire scores: European Organisation for Research and Treatment of Cancer Quality of Life Questionnaire-Core 30 (EORTC QLQ-C30), EuroQoL 5-Dimension 5-Level (EQ-5D-5L), Functional Assessment of Cancer Therapy-Colon Cancer (FACT-C), and Patient Global Impression of Change (PGIC)

- - A higher score on **EORTC QLQ-C30** global health status and the five functional assessments (physical, role, cognitive, emotional and social) represents better QoL while a higher value reflects worse symptoms for the nine items nausea and vomiting, pain, fatigue, dyspnea, insomnia, appetite loss, constipation, diarrhea and financial difficulties [1]
  - **EQ-5D-5L** consists of the EQ-5D descriptive system and the EQ visual analog scale (VAS) [2]
    - EQ-5D: The descriptive system has five dimensions (mobility, self-care, usual activities, pain/discomfort and anxiety/depression), each is rated according to a five-point verbal rating scale (VRS) (1. no problems, 2. slight problems, 3. moderate problems, 4. severe problems and 5. extreme problems)
    - EQ VAS: Records the patient’s self-rated health on a vertical VAS and used as a quantitative measure of health outcome, ranging from 0 (worst imaginable health state) to 100 (best imaginable state)
    - An EQ-5D-5L index value is obtained based on patient responses to the 5 dimensions and applying population-assessed weights (coefficients) to each set of responses that define a unique health state. Coefficients for China were used to compute the health state index as follows: index = 1 - _[coefficient of mobility + coefficient of self-care + coefficient of usual activities + coefficient of pain/discomfort + coefficient of anxiety/depression]
  - **FACT-C** consists of 36 items, presented on a five-point Likert scale, in four domains of well-being and the Colorectal Cancer Subscale (CCS). The FACT‑C Total score was computed by summing the FACT-G physical and functional domains and the CCS. Higher score reflects a better quality of life [3]
  - **PGIC** evaluates CRC symptoms since starting study intervention according to a seven-point verbal rating scale (1. very much improved, 2. much improved, 3. minimally improved, 4. no change, 5. minimally worse, 6. much worse, 7. very much worse [4]

**Analysis of Efficacy and Quality of Life Outcomes**

Clinical efficacy time-to-event endpoints (OS, DOR and TTR) were analyzed as the primary efficacy criterion for main analysis. QoL data were presented by treatment arm. The EORTC QLQ-C30 global health status score, EQ visual analog scale (VAS), and FACT-C total score were compared over time between the Doublet and Control arms using a repeated measurement analysis model, adjusted for baseline scores and the two stratification factors as covariates. Each model had an intercept term, a linear time trend term (in weeks), a term for treatment group, and a term for treatment-by-time interaction. The intercept and slope terms for time were random effects with an unstructured variance/covariance matrix. In addition, each observation was assumed to be measured with error and the error terms are independent of each other. All parameter estimates were obtained using restricted maximum likelihood estimation. The significance of the treatment-by-time interaction was evaluated first. The overall significance of the difference between the trajectories for the treatment arms was tested at a significance level of 0.05 (two-sided) within the model without the interaction. Adjusted means were provided for each treatment group as well as their 95% CI. Time to definitive deterioration, defined as at least a 10% worsening relative to baseline with no subsequent improvement, was assessed for both treatment arms and visualized using Kaplan-Meier curves. Median time to definitive deterioration, along with two-sided 95% CI, was provided. A Cox model was fitted with treatment arm and stratification factors as covariates to estimate the HR of the treatment effect, with corresponding 95% CI.

**References**

1. EORTC QLQ-C30 Scoring Manual. Available at: <https://www.eortc.org/app/uploads/sites/2/2018/02/SCmanual.pdf>. Accessed 22 November 2024.
2. EQ-5D-5L. Available at: <https://euroqol.org/>. Accessed 22 November 2024.
3. FACT-C website. Available at: <https://www.facit.org/measures/fact-c>. Accessed 04 October 2024.
4. PGI-C website. Available at: <https://eprovide.mapi-trust.org/instruments/patient-global-impressions-scale-change-improvement-severity>. Accessed 04 October 2024.
